# HIV Status Awareness, Partnership Dissolution and HIV Transmission in Generalized Epidemics

**DOI:** 10.1371/journal.pone.0050669

**Published:** 2012-12-17

**Authors:** Georges Reniers, Benjamin Armbruster

**Affiliations:** 1 Office of Population Research, Princeton University, Princeton, New Jersey, United States of America; 2 Department of Population Health, London School of Hygiene and Tropical Medicine, London, United Kingdom; 3 Northwestern University, Industrial Engineering and Management Sciences, Evanston, Illinois, United States of America; National Microbiology Laboratory, Canada

## Abstract

**Objectives:**

HIV status aware couples with at least one HIV positive partner are characterized by high separation and divorce rates. This phenomenon is often described as a corollary of couples HIV Testing and Counseling (HTC) that ought to be minimized. In this contribution, we demonstrate the implications of partnership dissolution in serodiscordant couples for the propagation of HIV.

**Methods:**

We develop a compartmental model to study epidemic outcomes of elevated partnership dissolution rates in serodiscordant couples and parameterize it with estimates from population-based data (Rakai, Uganda).

**Results:**

Via its effect on partnership dissolution, every percentage point increase in HIV status awareness reduces HIV incidence in monogamous populations by 0.27 percent for women and 0.63 percent for men. These effects are even larger when the assumption of monogamy can be relaxed, but are moderated by other behavior changes (e.g., increased condom use) in HIV status aware serodiscordant partnerships. When these behavior changes are taken into account, each percentage point increase in HIV status awareness reduces HIV incidence by 0.13 and 0.32 percent for women and men, respectively (assuming monogamy). The partnership dissolution effect exists because it decreases the fraction of serodiscordant couples in the population and prolongs the time that individuals spend outside partnerships.

**Conclusion:**

Our model predicts that elevated partnership dissolution rates in HIV status aware serodiscordant couples reduce the spread of HIV. As a consequence, the full impact of couples HTC for HIV prevention is probably larger than recognized to date. Particularly high partnership dissolution rates in female positive serodiscordant couples contribute to the gender imbalance in HIV infections.

## Introduction

A handful of studies have documented relatively high separation and divorce rates in serodiscordant couples [1]–[4], but no study has evaluated its implications for the spread of HIV. To the extent that partnership cessation has entered the debate on HIV prevention, it is usually considered a corollary of couples HIV Testing and Counseling (HTC) that ought to be minimized. In this contribution, we demonstrate by means of a mathematical model that partnership dissolution in serodiscordant couples can, under certain conditions, reduce the spread of HIV.

The role of serodiscordant partnerships for the propagation of HIV has generated considerable discussion in the HIV prevention literature. That debate intensified following studies claiming that about half (or more) of all HIV affected cohabiting couples are serodiscordant [5], [6]. Furthermore, one study claims that they are the locus for the majority of new infections [7]. The prominence of long-term serodiscordant partnerships for the spread of HIV is considered characteristic of mature generalized epidemics [8]. In contrast, during the early stages of an epidemic, new infections are believed to stem more often from contacts with high-risk groups.

These insights alone are not sufficient to present a strong case for policy interventions targeting serodiscordant couples, but they have been complemented by research that documents risk reduction behavior –in some instances corroborated by a decline in HIV incidence– following couples HTC [9]–[14]. One study suggested that couples HTC is more cost-effective than programs that target individuals [15]. Antiretroviral therapy (ART, for the HIV infected partner) [16], and pre-exposure prophylaxis (PrePEP, for the seronegative partner) have recently been added to the mix of possible interventions to reduce transmission within couples [17]. As a consequence, the policy support for couples HTC has strengthened [13], [18]–[21], but a consensus to turn it into an HIV prevention policy priority does not exist. Skeptics question whether the transmission in cohabiting serodiscordant couples indeed accounts for the majority of new infections [22], [23], and raise a number of practical and logistical concerns with scaling up couples HTC [24]. Many of those revolve around the low demand for, and uptake of couples HTC services [25]–[29]. As a result, couple counseling, while nominally supported by most agencies, is still not much more than a theoretically appealing intervention that has not yet fully materialized on its promise [30].

A final concern with couples counseling, or more generally, the disclosure of HIV positive status is usually referred to as the experience of negative life events. These include violence, rejection, stigmatization and divorce or separation [1], [2], [31]–[34]. Opinions differ over the magnitude and importance of these problems, but observers agree that HIV positive women are particularly vulnerable.

Without any intent to minimize the possible hardship for the men and women in these situations, we argue that elevated partnership dissolution rates in HIV status aware serodiscordant couples have an unintended advantageous effect in the sense that they limit the spread of HIV. This could have considerable public health implications because it means that the benefits of couples HTC may have been underappreciated to date. In this paper we support this claim by developing a simple compartmental model of the HIV infection status of individuals and couples. The model allows us to illustrate the effect of elevated partnership dissolution rates in HIV status aware couples on HIV incidence, and highlight the intermediary mechanism. Finally, we note that we merely offer “proof of principle” for a neglected hypothesis [35], and underscore that our model is not meant to precisely estimate the contribution of partnership dissolution to the prevention of HIV transmission.

## Methods

### Parameters

Our model focuses on the effects of elevated partnership dissolution rates among HIV status aware serodiscordant couples. HIV status awareness is directly related to HTC coverage rates and we will use both interchangeably in what follows. This implies a number of simplifying assumptions. In interpreting our results in terms of the effect of increasing HIV testing rates, for example, we ignore that HIV test results could be inaccurate or that a negative test loses value as time goes on. We also presume that all HIV test results are immediately disclosed to partners. To the extent that partners do not disclose their HIV status (and are not jointly counseled), our results will overestimate the impact of HTC on HIV incidence.We have made these choices for modeling simplicity and to maintain a focus on (mutual) HIV status awareness, partnership dissolution and HIV transmission.

We denote the serostatus of a couple by an ordered pair, with the serostatus of women listed first (e.g., −+ or F-M+). Let d(ij) be the dissolution rate of partnerships with status HIV ij. We also account for the fact that men and women in serodiscordant couples with information about each other's serostatus will take precautionary measures to prevent HIV transmission. This is the more conventional preventative effect of couples HTC described in the literature, and we let γ denote the relative risk of infection for those couples. The “monogamy” variant of our model requires some additional parameters, namely HIV prevalence and the fraction of individuals that are partnered in the absence of HTC.

To our knowledge, only two studies report detailed data on separation and divorce by the serostatus configuration of partners: Porter et al. [1] present data from a community-based randomized clinical trial of STD control for AIDS prevention (Rakai, 1994–1999). Men and women were tested for HIV and encouraged to participate in a post-test counseling session wherein HIV test results were returned. Out of a total of 6,433 married women at baseline, 4,507 were retrospectively linked to a spouse who was tested as part of the study. The receipt of HIV test results in the Rakai study was voluntary and not necessary for study participation (the uptake of post-test counseling is not reported), and the authors did not collect information about the HIV status disclosure to the partner. This contrasts with a study from Grinstead et al. [2] who report on a randomized HTC trial wherein around 560 couples participated (Nairobi, Dar-Es-Salaam & Port of Spain, 1995–1998). In this study, everyone in the intervention arm received their HIV test result and over 90 percent disclosed it to their partner (or were jointly counseled). For simplicity, we will assume that all HTC participants in both studies learned their HIV status and also relayed it to their partners.

The annual dissolution rates retrieved from both studies are presented in Table 1. The rates derived from Porter et al. are considerably lower than those derived from the Grinstead et al. and that could be due to differences in HIV status awareness in both studies (which in turn results from differences in the uptake of post-test counseling and the disclosure of HIV test results to partners). Another possible reason for the relatively low divorce rates reported in the Rakai study is that spouses were retrospectively linked. This linkage was less likely when the couple had divorced by the end of the follow up period. While the actual rates differ significantly, the ratios of the dissolution rates, d(ij)/d(--), are more similar in both studies. Dissolution rates also vary by partnership type (marriages versus informal sexual partnerships) and by the serostatus configuration of the couple (seroconcordant versus serodiscordant couples). In addition, female positive serodiscordant couples have the highest dissolutions rates, which corroborates the gender bias reported in the literature on negative life events following HTC.

**Table 1:** Annual partnership dissolution rates by HIV status of the couple, and other parameter values.

Annual dissolution rate, d(ij)	F-M−	F-M+	F+M−	F+M+
Porter et al. [1]	1%	2%	6%	5%
Grinstead et al. (marriages) [2]	5%	0%	33%	12%
Grinstead et al. (sex. partnerships) [2]	36%	62%	74%	38%

Ratio of dissolution rates, d(ij)/d(--)	F-M+	F+M−	F+M+
Porter et al. [1]	1.6	4.7	3.7
Grinstead et al. (marriages) [2]	-	6.7	2.3
Grinstead et al. (sex. partnerships) [2]	1.7	2.0	1.0

Other parameters	Baseline value (range)
HIV prevalence	10% (1%–15%)
Relative risk of infection for status-aware serodiscordant couples, γ	1/3 (0.1–1)
Fraction of individuals that are partnered in the absence of testing	2/3 (1/3–4/5)

Notes: The annual partnership dissolution rates are derived from Porter et al. [1] and Grinstead et al. [2]. From Porter et al. we take the 40-month dissolution probability from Table 5 and adjust for the fraction of women that are widowed and the duration to obtain an annual dissolution rate. More specifically, we divide by the fraction not widowed and then solve for the annual rate, a, such that 1-(1-a)^40/12^ = 1-(40-monthly rate). From Grinstead et al. we take the probability of a “break-up of a sexual relationship” and the probability of a “break-up of a marriage” after 7.3 months from Table 3 and adjust it similarly to obtain an annual dissolution rate. Neither of these studies contains sufficient detail to report the sample sizes on which these rates are based. The sample in Grinstead et al. is particularly small and it is unclear whether the numbers that are reported take the individual or couple as the unit of analysis. Carpenter et al. [3] also report dissolution rates by the serostatus of both spouses (with similar conclusions), but again based on a very small sample.

In what follows, we use the data from Rakai as our baseline because the study is population- rather than health facility-based and because the sample is much larger. Following Dunkle et al. [7], we set the baseline value for γ at 1/3, meaning that the transmission rate in HIV status aware serodiscordant couples is reduced by about 67 percent because individuals in such couples take precautionary measures for limiting HIV transmission. In the sensitivity analysis, we evaluate values for γ ranging from 0.1 to 1.

For the monogamy model developed below we also require estimates of HIV prevalence and an estimate of the fraction of individuals that are partnered in the absence of testing. Our baseline setting for HIV prevalence is 10 percent for both men and women, and in the sensitivity analysis we evaluate values from 1 to 15 percent. Our baseline assumption for the fraction of individuals partnered is 2/3 for both men and women. This value is an average of the fraction in a partnership at the time of the interview (both sexes, and both marriage and informal partnerships) for 19 recent African Demographic and Health Surveys (http://www.measuredhs.com). In the sensitivity analysis, we consider values ranging from 1/3 to 4/5.

### Mathematical model

To contain the complexity of the model, we assume that all partnerships are heterosexual; that the number of men and women are equal; and that HIV prevalence is equal for men and women. An individual's serostatus can be negative (−), positive and tested (+), or positive and untested (u). Untested individuals (irrespective of their HIV status) are assumed to behave like those who test negative, and thus we let d(i-) = d(iu) and d(-j) = d(uj) for all i and j.

Since we only examine a benefit of partnership dissolution, we make the conservative assumption that no serosorting takes place in the formation of new partnerships. Further, we assume that HTC is independent of HIV status itself. This assumption is probably violated at low HTC coverage rates [36], but increasingly realistic as the uptake of HTC increases.

We develop a mathematical model with two variants. Both variants are compartmental models with random mixing. The “concurrency” variant is stochastic and allows for multiple overlapping partnerships while the “monogamy” variant is deterministic and restricts individuals to at most one partner. The mathematical details of both model variants are given in Supporting Information S1. A key output of our model is the relative decrease in the rate of new infections:
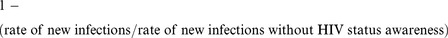



In the concurrency variant, we assume that the rate at which a person acquires a new partner does not depend on his or her existing number of partners. In this model variant, we further assume that the population is large and without constraints on partner availability. The concurrency model developed in Supporting Information S1 provides a formula for the rate of new infections. Specifically, each percentage point increase in the HIV status awareness or testing rate of men leads to a 1−γ d(−−)/d(−+) relative decrease in the rate of new infections among women. Mathematically,
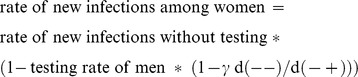


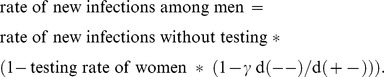



Our model calculates the fraction of the total population in various relationship types (single, seroconcordant, and serodiscordant) and, ultimately, the HIV infection rates that they produce. As we show, these outcomes are highly dependent on HTC coverage rates. The model variant assuming monogamy does not have an analytical solution but we can solve it numerically.

## Results

**Figure 1:**
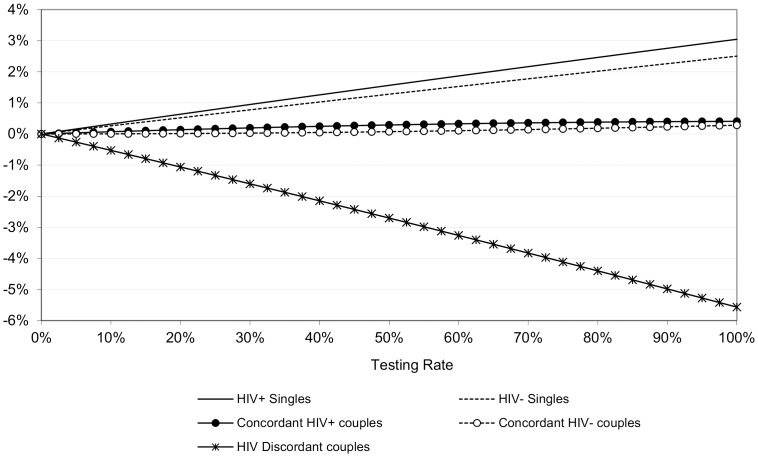
Estimated change in the percentage of the total population in various relationship types at different HTC coverage rates (monogamy model with baseline parameter values).

**Figure 2:**
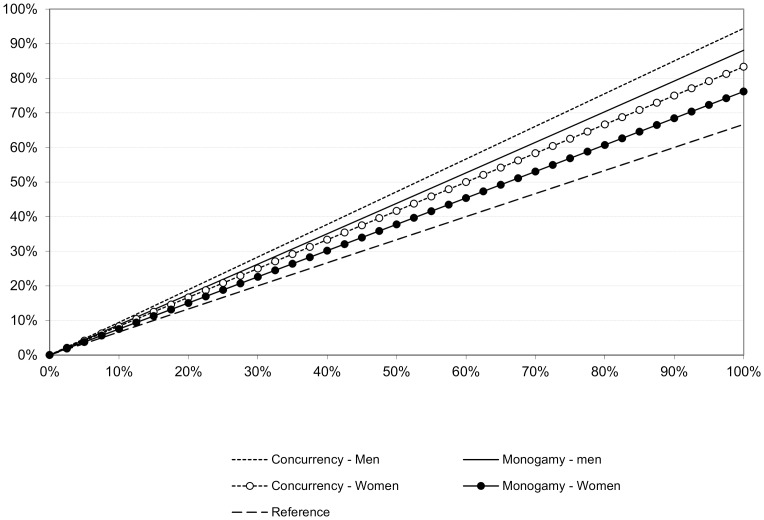
Relative Percentage decline in new HIV infections by HTC coverage and scenario.

We first show how increases in HTC coverage rates or HIV status awareness change the (joint) HIV status distribution of both singles and couples in the population (monogamy model only, Figure 1). Most importantly, the fraction of serodiscordant couples declines, and that is matched by increases in the fractions of HIV positive and HIV negative singles as well as the share of seroconcordant partnerships. These changes are close to linear in the HTC coverage. Taken together, these changes in relationship status distributions will have beneficial effects for HIV prevention because they reduce HIV negative individuals' exposure to the virus. This is further explored in Figure 2, which summarizes the reduction of new infections in the random mixing models with concurrency and monogamy and at different levels of HTC coverage or HIV status awareness.

The reference scenario in Figure 2 assumes that partnership dissolution rates are not dependent on the serostatus configuration of the couple, but accounts for the fact that men and women in HIV status aware serodiscordant couples make behavioral adjustments to limit HIV transmission. We can derive three important conclusions from the graph. First, elevated separation and divorce rates in couples where at least one spouse is HIV positive reduces the epidemic propagation beyond the reduction in new infections attributable to behavioral adjustments. More precisely, assuming monogamy, the elevated dissolution rates in HIV status aware couples with at least one infected partner increase the protective effect of HTC on HIV incidence by 13 and 32 percent for women and men, respectively (at a testing rate of 50%). Without the restriction of monogamy, the elevated dissolution rates enhance the protective effect of HTC by 25 percent for women and 42 percent for men. Both effects combined (dissolution and behavioral adjustments within serodiscordant couples) reduce HIV incidence by 38 to 47 percent at an HTC coverage rate of 50 percent, and by 76 to 94 percent when everyone is aware of one's own as well as one's partner's HIV status.

Second, partnership dissolution has a substantially larger downward effect on new infections among men compared to women, and the reason is that the empirical dissolution rates are considerably higher in female positive compared to male positive serodiscordant couples. Third, the protective effect of partnership dissolution is larger in populations with partnership concurrency compared to populations where strict monogamy is the norm. This is due to a second order effect whereby elevated divorce rates cause the HIV prevalence among singles to rise. Under monogamy, these singles are drawn back into the marriage market as new unions are formed and that mitigates the protective dissolution effect. In our partnership concurrency scenario there are no restrictions on the pool from which the new partners come, and because the HIV prevalence is lower in the total population compared to the subgroups of singles, the relative share of serodiscordant couples will be lower in a scenario that allows for partnerships concurrency. The random mixing model with concurrency that is presumed here may not be very realistic, but it is interesting because it highlights that constraints in partnership markets interact with other sexual network attributes to inflate or moderate the dissolution effect on HIV incidence.

**Figure 3:**
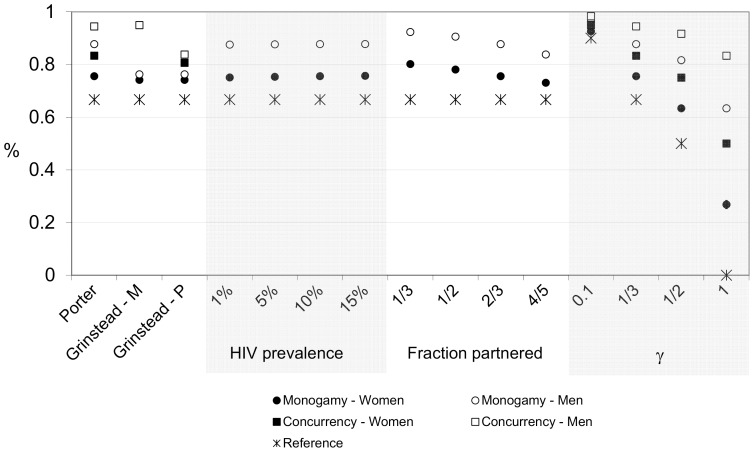
Sensitivity analysis: relative percentage decline in new HIV infections for each percentage point increase in HIV status awareness. Notes: Grinstead – M = Marriages; Grinstead – P = sexual partnerships. The reference scenario only accounts for behavioral adjustments to limit transmission within serodiscordant couples (γ = 1/3); the other scenarios demonstrate the total effect of HTC through both behavior change and partnership dissolution. The estimate of the effect of HTC for females in the concurrency model with the parameters for marriages from Grinstead et al. is not shown because no F-M+ marriages dissolved in that study, leading to a division by zero in our calculations. The actual percent decline in new infections varied slightly with HIV testing coverage but is always within 0.02 of the value shown. The first scenario of panel 1, the third scenario of panel 2, the third scenario of panel 3 and the second scenario of panel 4 are the baseline scenario and are thus identical. The concurrency model is not dependent on HIV prevalence or the fraction partnered and its estimates have been omitted for those scenarios.

In Figure 3, we show the results of a sensitivity analysis. Since the relationship between the testing rate and the decrease in new infections is close to linear (and exactly linear for the concurrency model), we only show the ratio or the decrease in new infections for each percentage point increase in the testing rate. In the sensitivity analysis, we vary (a) the dissolution rates as observed in the two studies, (b) HIV prevalence, (c) the fraction of the population in partnerships (in the absence of testing), and (d) the value of γ.

From the first panel in Figure 3, we learn that the dissolution parameter settings from Grinstead et al. produce smaller gender differences in HIV incidence than those derived from Porter et al, and that appears to be the case for both marriages and informal partnerships. To put these estimates in context, we reiterate that the parameter estimates from Grinstead et al. are based on a very small sample. From the second panel, we learn that the protective dissolution effect is virtually independent of the HIV prevalence in the population. The fraction partnered is a more important mediating factor (panel 3): as the fraction of individuals in partnerships goes up, the high dissolution rates among couples with at least one seropositive partner will have a smaller effect in limiting new infections and the protective effect of HTC decreases. In the last panel, γ = 1 represents the extreme case where serodiscordant couples do not make any other behavioral adjustments for reducing HIV transmission. In that scenario HIV incidence is reduced by 0.27 and 0.63 percent for every percentage point increase in the HIV testing rate under the scenario of strict monogamy and for women and men respectively. In a population where new partnerships are not restricted to singles, these effects are even larger: 0.50 percent for women and 0.83 percent for men. As γ approaches 0, the transmission ceases in HIV status aware serodiscordant couples and the advantageous effect of elevated dissolution rates on the epidemic potential disappears.

## Conclusion

Our model suggests that elevated partnership dissolution rates in serodiscordant couples reduce HIV incidence. In the absence of other (behavioral) adjustments to reduce transmission in HIV status aware serodiscordant couples (γ = 1), these effects are quite large: every percentage point increase in HIV testing coverage reduces HIV incidence by 0.27 percent for women in populations practicing monogamy to 0.83 percent among men in populations where there are no restrictions on the choice of new partners. Of course, individuals in HIV status aware serodiscordant couples can be expected to take precautionary measures to prevent HIV transmission (e.g., condom use, ART for the infected partner and PrePEP for the uninfected partner) and that reduces the net effect of divorce and separation.

Our results also indicate that higher dissolution rates in female compared to male positive serodiscordant couples contribute to the gender imbalance in the sex ratio of HIV infections. The reason is that HIV negative women will spend more time in serodiscordant relationships than men.

We have tied our discussion of the effects of partnership dissolution in serodiscordant couples with efforts to increase HTC because that is the policy area where it is most relevant. Whereas it might not be desirable to counsel serodiscordant couples to separate, the (unintended) dissolution effect on epidemic propagation deserves inclusion in cost-benefit analyses of HTC. We also stress that men and women do not necessarily have to be aware of each other's HIV status for such a pattern to arise. Both qualitative and quantitative studies from Malawi, for example, have described in considerable detail that individuals will act on the suspicion of HIV infection and correlated behaviors (e.g., adultery) to initiate a divorce [37]–[40]. In combination with our modeling results, this last observation points at the possible importance of pro-active partnership dissolution for understanding declines in HIV incidence and prevalence in sub-Saharan Africa that occurred before the large-scale accessibility of HTC services. Of course, decision-making on the basis of imperfect information may lead seroconcordant couples to dissolve in situations where they would have stayed intact if they had access to accurate HIV status information. It is easy to show as an extension of our analysis that elevated dissolution rates in seroconcordant couples will increase HIV incidence. Once again, this underscores the benefits of couples HTC.

As with any modeling project, the limitations of this study stem from a number of simplifying assumptions. We have discussed the most important of these in the methods section, but wish to reiterate that our model, because of its static nature, does not account for long-term changes in sexual networks or the social position of women induced by higher divorce and separation rates (e.g., changes in the relative importance of long-term versus short term partnerships in the population, or, changes in the demand or supply of sex for money). Similarly, we have chosen not to model mortality (widowhood), ART coverage, or variability in viral load by duration since HIV acquisition, and we do not have the data to account for HIV status disclosure to partners in greater detail. Some of these limitations can be remedied by the collection of better data, starting with more detailed reports on joint couples HTC and HIV status disclosure to partners following individual HTC and better data on separation and divorce following HTC.

## Supporting Information

Figure S1 **Flow model for the variant assuming monogamy.**
(TIF)

Supporting Information S1 **Mathematical model.**
(DOCX)
