# Supplementary material for: HIV Status Awareness, Partnership Dissolution and HIV Transmission in Generalized Epidemics
Source: PLoS One. 2012 Dec 17;7(12):e50669. doi: 10.1371/journal.pone.0050669 (PMC3524232; doi:10.1371/journal.pone.0050669)
Supplement: Supporting Information S1 — Mathematical model. (DOCX) [file pone.0050669.s002.docx]

**Supporting information: Mathematical model**

Let t(f) and t(m) be the proportion of women and men who know their HIV status (i.e., the testing rates); let p(m) and p(f) be the HIV prevalence for men and women; let the number of men and women both equal n; let n(ij) be the number of partnerships where the woman has serostatus i and the man has serostatus j. An individual’s serostatus can be negative (-), positive and tested (+), or positive and untested (u). Tested and untested HIV negative individuals are assumed to behave alike and are not distinguished. We assume that untested individuals act like those who test negative, and thus we let d(i-)=d(iu) and d(-j)=d(uj) for all i and j. We let β(f) and β(m) be the annualized HIV acquisition rates among HIV negative women and men with seropositive partners, and thus the annual number of new infections among women is β(f) (γn(-+)+n(-u)), and β(m) (γn(+-)+n(u-)) for men. Our goal in the two model variants below is to determine how testing changes γn(-+)+n(-u) and γn(+-)+n(u-).

**Concurrency model variant**

This model makes use of the following lemma: if individuals form new partnerships at a rate λ and break-up with each existing partner at a rate μ, then in steady-state the number of partners is given by a Poisson distribution with mean λ/μ. To prove this we first note that the number of partners follows a birth-death Markov process, and then we solve for the steady-state distribution.

For various types of relationships we describe their number, their partnership formation rates, and their dissolution rates. We let λ be the overall partnership formation rate, and we let x(i) be the fraction of eligible pairs of type i, for example: x(-u)=(1-p(f))p(m)(1-t(m)). Hence each individual forms relationships of type i at a rate of λ x(i). We let d(i) be the dissolution rate for such relationships. Thus the average number of partnerships of type i is n(i)=n λ x(i)/d(i). Hence γ n(-+)+n(-u) = n λ (1-p(f)) p(m) [γ t(m)/d(-+) + (1-t(m))/d(-u)].

This simplifies to:

γ n(-+)+n(-u)=n λ (1-p(f)) p(m)/d(--) [1 - t(m)(1-γ d(--)/d(-+))]

Similarly, we also have:

γ n(+-)+n(u-)=n λ (1-p(m)) p(f)/d(--) [1 - t(f)(1-γ d(--)/d(+-))]

These expressions demonstrate the effect of HTC for both men and women: an increase in the testing rate among men causes a relative decline in the number of male positive serodiscordant couples and therefore also a decline in the number of new infections among women. The magnitude of that effect depends on the ratio γ d(--)/d(-+). The same holds for women.

**Monogamy model variant**

As the name suggests, this variant of the model assumes that individuals have at most one partner. We expand the previous notation to allow for the status u, denoting a negative or untested individual, for example n(+u)=n(+-)+n(+u), and —since the behavior of negative and untested individuals is assumed to be the same— n(+-)=n(+u) (1-p(m))/(1-p(m)t(m)). We also expand the notation by having 0 refer to no partner, that is n(i0) is the number of single women with serostatus i. The fraction of single men that are positive and tested is defined as a(m)=n(0+)/(n(0+)+n(0u)). We define a(f) similarly. As in the previous model, λ is the overall rate that an individual forms a partnership and d(i) is the rate at which a partnership of type i dissolves. Figure S1 illustrates the various states and the flows among them for both men and women and the corresponding system of ordinary differential equations (ODEs). This deterministic flow model can also be interpreted as a stochastic Markov process where n(ij)/n is the fraction of time that a woman in state i has a partner in state j (or a man in state j has a partner in state i).

Setting the right hand sides to zero, we obtain the following equations describing the steady-state:

n(i0) λ (1-a(m))=n(iu) d(i-) and n(i0) λ a(m) = n(i+) d(i+) for i={u,+}

n(0j) λ (1-a(f))=n(uj) d(-j) and n(0j) λ a(f) = n(+j) d(+j) for j={u,+}.

The HIV awareness level and the fact that there are a total of *n* men and *n* women provides us with the following additional equations:

n(0+)+n(u+)+n(++)=t(m)p(m)n and n(0u)+n(uu)+n(+u)=(1-t(m)p(m))n

a(m)=n(0+)/(n(0+)+n(0u)) and a(f)=n(+0)/(n(+0)+n(u0)).

Together, this system of equations can be solved numerically, but in order to do that we need an estimate of the partnership formation rate λ. We choose λ using an estimate of the fraction of individuals who are partnered in the absence of testing. Without any testing, the positive compartments are empty, and thus the estimated fraction of men and women who are partnered is n(uu)/n. Without testing it also follows that a(m)=a(f)=0, and thus, the steady-state equation for single, untested women is $0=d\left( \underline{u}- \right)n\left( \underline{u}\underline{u} \right)-\lambda n\left( \underline{u}0 \right)$. Note that n(uu)+n(u0)=n and d(u-)=d(--). Hence it follows that n(uu)/n = λ/(λ+d(--)), allowing us to set λ using our estimate.
